# Reduced morbidity and mortality of cGVHD in patients who received treatment with mesenchymal stromal cells for steroid-resistant aGVHD: long-term follow-up of a randomized phase 3 trial

**DOI:** 10.1186/s40164-025-00687-8

**Published:** 2025-07-09

**Authors:** Ke Zhao, Ren Lin, Zhiping Fan, Zhen Li, Xiaoyong Chen, Li Xuan, Fen Huang, Na Xu, Xiuli Wu, Shaohua Chen, Jing Sun, Xi Zhang, Jianyu Weng, Yonghua Li, Yuhua Li, Dongjun Lin, Danian Nie, Shunqing Wang, Xiaojun Xu, Xiaohui Zhang, Yangqiu Li, AP Xiang, Yu Wang, Qifa Liu

**Affiliations:** 1 Department of Hematology, Nanfang Hospital, Southern Medical University, 1838 Guangzhou Blvd North, Guangzhou, 510515 China; 2 Clinical Medical Research Center of Hematology Diseases of Guangdong Province, Guangzhou, 510515 China; 3 Center for Stem Cell Biology and Tissue Engineering, Sun Yat-Sen University, Guangzhou, 510080 China; 4 Institute of Hematology, Medical College, Jinan University, Guangzhou, 510632 China; 5 Medical Center of Hematology, Xinqiao Hospital, Army Medical University, Chongqing, 400037 China; 6 Department of Hematology, Guangdong Academy of Medical Sciences, Guangdong Provincial People’s Hospital, Southern Medical University, Guangzhou, 510080 China; 7 Department of Hematology, General Hospital of Southern Theatre Command, Guangzhou, 440104 China; 8 Department of Hematology, Zhujiang Hospital, Southern Medical University, Guangzhou, 510282 China; 9 Department of Hematology, The Seventh Affiliated Hospital of Sun Yat-Sen University, Shenzhen, 510630 China; 10 Department of Hematology, The Third Affiliated Hospital of Sun Yat-Sen University, Guangzhou, 510630 China; 11 Department of Hematology, Sun Yat-Sen Memorial Hospital of Sun Yat-Sen University, Guangzhou, 510120 China; 12 Department of Hematology, Guangzhou First People’s Hospital, Guangzhou Medical University, Guangzhou, 510180 China; 13 Department of Hematology, Peking University People’s Hospital, Beijing, 100044 China; 14 Department of Hematology, Beijing Lu Daopei Hospital, Beijing, 100176 China

**Keywords:** Mesenchymal stromal cell, Chronic graft-versus-host disease, Acute graft-versus-host disease, Allogeneic hematopoietic stem cell transplantation, Thymus

## Abstract

**Background:**

Our open-label, multicenter, randomized, phase 3 trial showed that the incidence and severity of chronic graft-versus-host disease (cGVHD) reduced in steroid-resistant acute graft-versus-host disease (aGVHD) patients who underwent mesenchymal stromal cells (MSCs) treatments, but survival benefit was not received. Here, we present a post-hoc analysis of the 5-year follow-up to explore long-term survival and its underlying mechanism.

**Methods:**

This long-term follow-up trial included steroid-resistant aGVHD patients, who were randomly assigned (1:1) to receive MSCs (MSC group) (1 × 10^6^ cells/kg once weekly for 4 consecutive weeks, 8 doses at most) or without MSCs treatment (control group). For this updated analysis, the 5-year endpoints were cumulative incidence of cGVHD, overall survival, cGVHD-free, relapse-free survival (CRFS), and relapse. To explore the mechanism, We investigated the changes in T, B cells, and signal joint T cell receptor excision DNA circles (sjTRECs).

**Results:**

Between September 2014 and March 2019, 198 patients were randomly assigned to the MSC group (*n* = 99) or the control group (*n* = 99). Extended follow-up showed the lower 5-year cumulative incidence of cGVHD (42.0% [95%CI 32.2–51.5] vs. 67.1% [55.6–76.3]; hazard ratio [HR] 2.19, 95%CI 1.47–3.27; *P* < 0.001), improved 5-year overall survival (60.4% [50.8–70.0] vs. 41.7% [31.9–51.5]; 0.63, 0.42–0.94; *P* = 0.023), CRFS (33.9% [24.5–43.3] vs. 20.9% [12.9–28.9]; 0.67, 0.48–0.93; *P* = 0.017) and no increase on relapse (13.6% [7.6–21.3] vs. 16.0% [9.5–23.9]; 1.24, 0.60–2.56; *P* = 0.568) for patients in MSC group compared with the control group. Clinical improvement of MSCs was accompanied by significant increases in regulatory T cells, CD4 + CD45RA + CD31 + naïve T, CD19 + CD27 + IgD- memory B cells, and sjTRECs.

**Conclusions:**

With extended follow-up, MSCs reduced the morbidity of cGVHD in aGVHD patients and improved overall survival and CRFS. Mechanistically, MSCs reduced cGVHD by thymus pathway.

**Trial registration:**

clinicaltrials.gov identifier: NCT02241018. Registration date: 16 September 2014, https://clinicaltrials.gov/ct2/show/NCT02241018.

**Supplementary Information:**

The online version contains supplementary material available at 10.1186/s40164-025-00687-8.

## Background

Graft-versus-host disease (GVHD) is a serious complication contributing to mortality after allogeneic hematopoietic cell transplantation (allo-HSCT). It is categorized into two distinct types, acute GVHD (aGVHD) and chronic GVHD (cGVHD) [1–4]. Despite marked improvements that have been made in GVHD prophylaxis, aGVHD occurs in 30–70% of patients undergoing allo-HSCT, whereas 30–50% of patients develop cGVHD [3–7]. Since cGVHD often evolves from aGVHD, aGVHD is an important risk factor for predicting the incidence and severity of cGVHD [8, 9]. Almost 80% of cGVHD patients have a history of aGVHD, and these patients have a worse prognosis than de novo cGVHD [9, 10]. However, the pathologic mechanism whereby aGVHD proceeds to cGVHD remains poorly understood. The possible mechanism is that cGVHD results from impaired negative selection in the thymus caused by alloreactive T cells during aGVHD [11–14]. Therefore, the important strategy for GVHD is modulating aGVHD development and preventing aGVHD from evolving to cGVHD.

Nowadays, a growing number of studies demonstrate that mesenchymal stromal cells (MSCs) are effective for the prophylaxis and treatment of aGVHD [15–24]. Thus, MSCs are recommended as evidence level A-II for aGVHD second-line treatment [25]. In the aspect of cGVHD, few published clinical studies exhibit that MSCs are also effective for cGVHD prophylaxis and treatment [26–30]. Recently, two randomized controlled clinical trials by Zhang et al. described encouraging cGVHD prophylactic efficacy after MSCs infusion in the early stage (45 days to 81 days) or later stage (100 days) after haploidentical HSCT [27, 30]. In our two prospective short-term follow-up studies, we found that MSCs reduced the incidence and severity of cGVHD in aGVHD patients who received MSCs treatment. However, no definitive conclusions could be made about survival benefits [17, 18] The follow-up time of our previous study is not enough for evaluating the 2-year cumulative incidence of cGVHD. Meanwhile, the mechanism of MSCs reducing cGVHD has not been elucidated. In this article, we add a 3-year follow-up to our previous phase 3, randomized, controlled study to further verify this interesting discovery, and explore the long-term survival benefit and the mechanism that MSCs reduce the morbidity and mortality of subsequent development of cGVHD in aGVHD patients.

## Methods

### Study design and patients

This long-term, post-hoc, follow-up analysis of a multicentre, open-label, randomized trial was done in nine centers in China. Details about the original study design and participants had been previously published [17]. Briefly, the patients were eligible for inclusion if they were aged 14 to 65 years and diagnosed with steroid-resistant (SR) aGVHD. The diagnosis of aGVHD was according to the literature criteria established by the Mount Sinai Acute GVHD International consortium group [31]. SR aGVHD was defined as aGVHD worsening after 3 days of therapy onset with ≥ 2 mg/kg/day of methylprednisolone or equivalent, or failure to improve after 7 days of treatment initiation; or treatment failure during steroid taper [32, 33]. Patients were excluded from the study if aGVHD occurred due to tapering/discontinuing immunesuppressors or donor lymphocyte infusion (DLI) for prevention/treatment of primary disease relapse, received more than one previous treatment for SR aGVHD except for steroids before randomization, had uncontrolled infections, active visceral hemorrhage, or severe concomitant conditions not suitable for the trial. The ethics committee review boards of the participating centers approved the study protocol, and all patients (or their guardians) provided written informed consent before inclusion in accordance with the Declaration of Helsinki.

### Randomization and masking

Details about randomization and masking had been previously published [17]. Eligible patients were randomly assigned (1:1) to MSC or non-MSC (control) group. We did randomization using permuted blocks (block size four) and we implemented it through an interactive web-based response system, which was independent of study site staff and investigators. Randomization codes were generated independently of the study by an independent statistician. Study treatment was not masked to investigators or participants. Study staff who evaluated the outcomes and analyzed the data were masked to treatment assignments.

### Procedures

MSCs were manufactured and provided by the Center for Stem Cell Biology and Tissue Engineering, Sun Yat-Sen University. MSCs were obtained from fresh BM of unrelated, HLA-mismatched, third-party donors after written informed consent. Isolation, ex vivo expansion, and identification of MSCs were performed in accordance with our previous publication [18, 34]. Cells were harvested at passages 4 to 5, and fresh meeting release criteria MSCs were shipped to the clinical sites in 100 ml saline with a continuous temperature monitoring device at 4 °C.

Details about the procedures had been previously published [17]. For patients assigned to the control group, basiliximab and calcineurin inhibitor were administered as “specified standardized second-line therapy” in the first cycle (from the initial treatments to day 28). Steroids were tapered after two doses of basiliximab and other immunosuppressive agents were allowed after one cycle in no-response (NR) patients by the attending physician. NR patients evaluated at day 28 in the control group could choose to receive MSCs treatment based on their voluntary principle. Patients assigned to the MSC group, not only received treatments as the control group but also received MSCs treatment. MSCs were administered intravenously at a dose of 1 × 10^6^ cells/kg once weekly for 4 consecutive weeks as a cycle. Further administration of MSCs was based on the response of MSCs evaluated at day 28. Complete response (CR) and NR patients discontinued MSCs treatment, while partial response (PR) patients continued to receive MSCs until aGVHD showed CR or MSCs had been infused for 8 doses.

### Outcomes

Details about the outcomes of the original trial had been previously published [17]. The primary endpoint was the OR at day 28, and the secondary endpoints were the durable overall response (OR) at day 56, failure-free survival, 3-year overall survival (OS), 2-year cumulative incidence of cGVHD, 3-year cumulative incidence of relapse, 3-year non-relapse mortality (NRM) and adverse events (up to 180 days after study treatments).

For this updated post-hoc analysis, the 5-year endpoints were cumulative incidence of cGVHD; OS; cGVHD-free, relapse-free survival (CRFS); relapse; NRM. cGVHD were graded according to published guidelines [35]. OS was defined as the time from randomization to death from any cause. CRFS included survival without the development of cGVHD plus disease relapse or progression or death. Relapse was defined as either reappearance of leukemic blasts in the peripheral blood or at least 5% blasts in the bone marrow aspirate or biopsy specimen not attributable to any other cause, or reappearance or new appearance of extramedullary leukemia. NRM was defined as death from any cause not subsequent to relapse.

### Exploratory analyses

Exploratory analyses included the effect of MSCs on lymphocyte subtypes and signal joint T cell receptor excision DNA circles (sjTRECs). Venous blood samples were collected before study treatments and at 1, 2, 3, 6, 9, and 12 months after study treatments. For details of peripheral blood mononuclear cell separation and staining with florescence-coupled monoclonal antibodies, refer to Supplementary Methods. T lymphocyte subsets (CD3^+^, CD3^+^CD4^+^, CD3^+^CD8^+^, CD4^+^CD25^+^Foxp3^+^, CD4^+^CD45RA^+^CD31^+^) and B lymphocytes (CD19^+^, CD19^+^CD27^−^IgD^+^, CD19^+^CD27^+^IgD^−^) in peripheral blood were analyzed by flow cytometry. Quantification of sjTRECs was analyzed by real-time PCR according to methods described previously [36]. A duplex vector that included a fragment of the dRec-𝝍Ja sjTREC and a fragment of the RAG2 was used to precisely determine the percentage of cells carrying sjTREC. The amplification was performed on MJ Research DNA Engine Opticon 2 PCR cycler (Bio-Rad, Hercules, CA, USA).

### Statistical analysis

Information about the sample size for the original trial had been previously published [17]. The sample size was established to show an increase from 70 to 90% in the OR rate at day 28, with a two-sided significance level of 0.05 and a power of 80%. After adjusting for a 5% dropout rate, the total planned sample size was 196 patients (98 in each group). We intentionally recruited more patients at the beginning of the study because of concerns about a delay in primary events compared with initial expectations.

The statistical analysis for this updated analysis was done in the intention-to-treat (ITT) population on December 31, 2023. The ITT population was defined as all randomly assigned patients, which was the basis for the analysis of survival, infection, and relapse. The cumulative incidence of cGVHD was performed in the modified ITT (mITT) population, which excluded patients who received DLI as a prevention/treatment for relapse. All analyses in this updated study were exploratory by definition because they were not specified in detail in the original study protocol. We compared categorical data using the χ2 test and numerical data using the Mann-Whitney U test. We estimated OS and CRFS using the Kaplan-Meier method and compared Kaplan-Meier curves using the log-rank test. The hazard ratios (HRs) and 95% confidence intervals (CIs) were estimated using the Cox proportional hazards model. Cumulative incidence of cGVHD, relapse, and NRM were evaluated as competing events. Competing risks for cGVHD included death without cGVHD and relapse. Relapse was a competing risk for NRM, and NRM was a competing risk for relapse. We used the Fine and Gray model to compare the cumulative incidences in the presence of a competing risk [37]. Relative levels of lymphocyte subtypes and sjTREC were compared using the Wilcoxon rank-sum test. All statistical tests were two-tailed with a significance level of 0.05. We used SPSS (version 21.0) and R (version 3.3.0) for data analysis.

## Results

### Patients

**Fig. 1:**
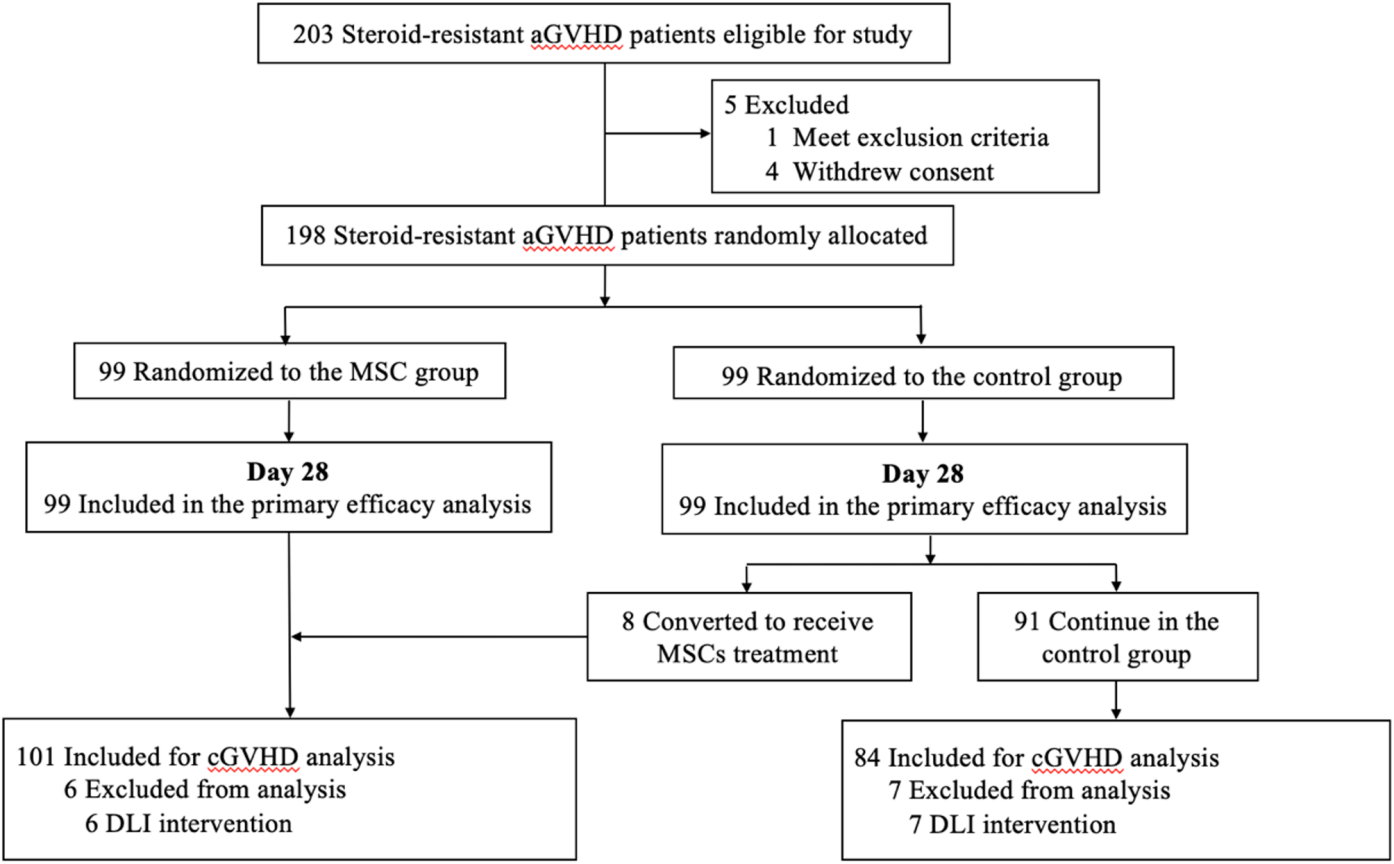
Trial profile

Detailed information regarding patient characteristics had been previously published [17]. Between September 2014 and March 2019, 198 patients with SR aGVHD were enrolled in the trial: 99 patients were randomly allocated to the MSC group and 99 patients to the control group (Fig. 1). The analysis of cGVHD consisted of 101 patients in the MSC group (99 per-protocol patients plus 8 patients with protocol crossovers, minus 6 patients receiving DLI as prevention or treatment for relapse), and 84 patients in the control group (99 per-protocol patients minus 8 patients with protocol crossovers and 7 patients receiving DLI as prevention or treatment for relapse). The baseline demographic, GVHD, transplantation and disease-related characteristics of patients were well-balanced between groups (Table 1). Of 198 enrolled patients, 73 patients (36.9%) had grade II aGVHD, 85 (42.9%) had grade III, and 40 (20.2%) had grade IV aGVHD.

**Table 1:** Baseline, disease, transplantation, and GVHD characteristics of patients in two groups

Variable	MSC group No. (%)	Control group No. (%)	*P*
No. of patients	99	99	
Age, median (range), years	28 (16–59)	29 (16–57)	0.680
Sex			0.385
Male	62 (62.6%)	56 (56.6%)	
Female	37 (37.4%)	43 (43.4%)	
Disease			0.129
AML	39 (39.4%)	49 (49.5%)	
ALL	45 (45.5%)	43 (43.4%)	
Others^*^	15 (15.2%)	7 (7.1%)	
MDS	6 (6.1%)	3 (3.0%)	
CML	3 (3.0%)	1 (1.0%)	
MM	1 (1.0%)	0	
NHL	0	1 (1.0%)	
Leukemia	5 (5.1%)	2 (2.0%)	
Disease status at transplant			0.524
CR	63 (63.6%)	70 (70.7%)	
PR	6 (6.1%)	6 (6.1%)	
NR	30 (30.3%)	23 (23.2%)	
Transplant modality			1.000
HLA-matched sibling donor	44 (44.4%)	44 (44.4%)	
HLA-matched unrelated donor	7 (7.1%)	7 (7.1%)	
HLA-haploidentical donor	48 (48.5%)	48 (48.5%)	
Conditioning			0.200
Bu-based	51 (51.5%)	42 (42.4%)	
TBI-based	48 (48.5%)	57 (57.6%)	
Donor source			0.567
PBSC	53 (53.5%)	57 (57.6%)	
PBSC + BM	46 (46.5%)	42 (42.4%)	
GVHD prevention			0.886
CsA + MTX or CsA + MTX + MMF	42 (42.4%)	43 (43.4%)	
CsA + MTX + MMF + ATG	57 (57.6%)	56 (56.6%)	
Grade of aGVHD			0.771
Grade 2	36 (36.4%)	37 (37.4%)	
Grade 3	41 (41.4%)	44 (44.4%)	
Grade 4	22 (22.2%)	18 (18.2%)	
No. of aGVHD involved organs			0.589
1	25 (25.3%)	29 (29.3%)	
2	46 (46.5%)	48 (48.5%)	
3	28 (28.3%)	22 (22.2%)	
aGVHD involved organs			
Skin	73 (73.7%)	63 (63.6%)	0.728
Liver	43 (43.4%)	46 (46.5%)	
GI	85 (85.9%)	82 (82.8%)	
Onset of aGVHD median days (range)	30 (14–132)	28 (16–124)	0.736

### cGVHD

**Fig. 2:**
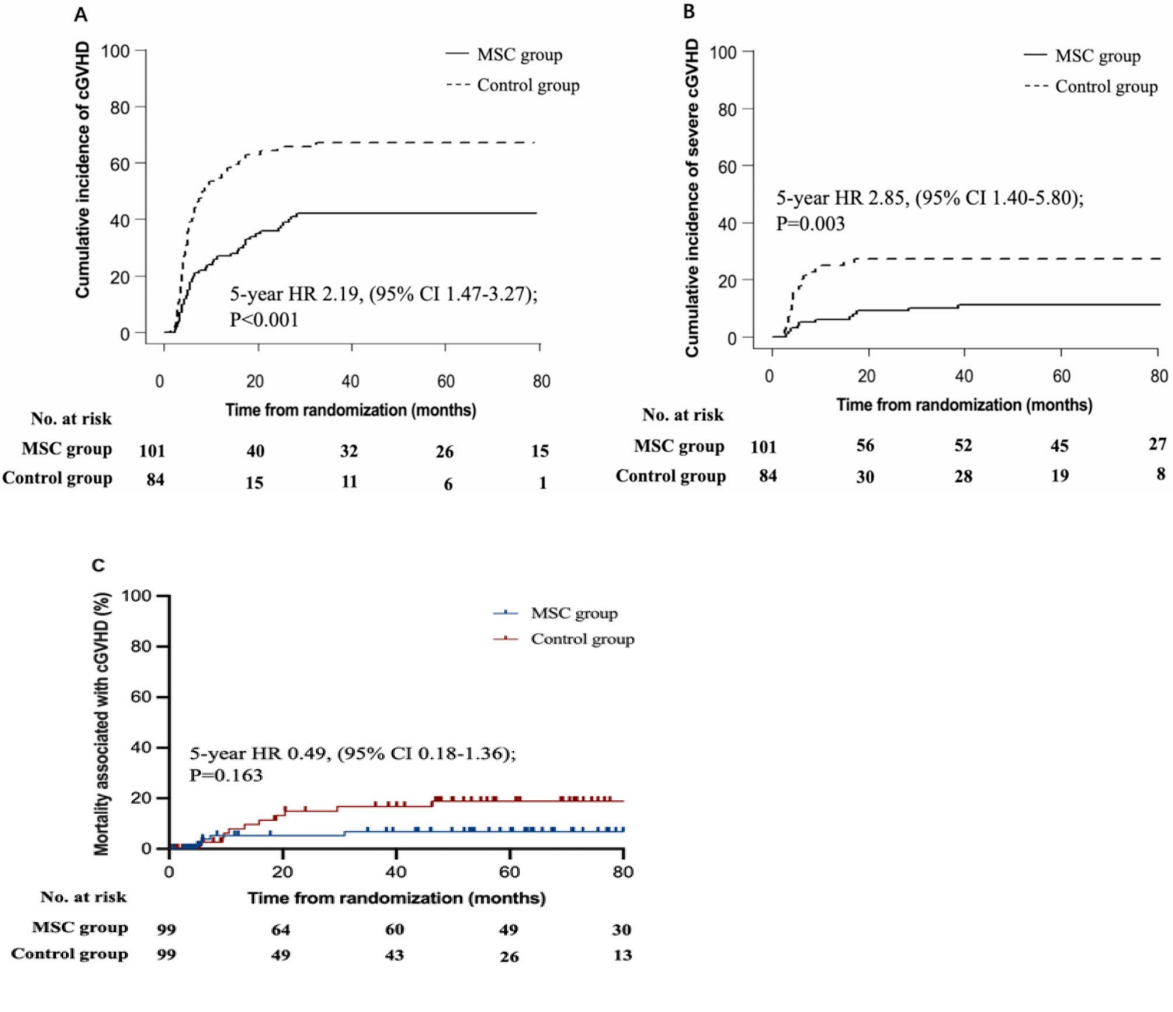
Morbidity and mortality of cGVHD. Cumulative incidence of overall chronic graft-versus-host disease (cGVHD) (**A**), severe cGVHD (**B**), and mortality associated with cGVHD (**C**)

A total of 185 patients enrolled in the cGVHD analysis including 101 in the MSC group and 84 in the control group. The median time from transplantation to diagnosis of cGVHD was 7.00 months (2.43–28.47) in the MSC group and 5.10 months (1.47–32.53) in the control group. The maximum overall severity of cGVHD was mild in 10 patients (9.9%), moderate in 21 (20.8%), and severe in 11 (10.9%) in the MSC group, whereas mild in 7 patients (8.3%), moderate in 30 (35.7%), and severe in 20 (23.8%) in the control group. The 5-year cumulative incidence of overall cGVHD was 42.0% (95% CI 32.2–51.5) for patients in MSC group and 67.1% (55.6–76.3) for those in the control group (HR 2.19, 95% CI 1.47–3.27; *P* < 0.001; Fig. 2A), and the 5-year cumulative incidence of severe cGVHD was 11.0% (5.8–18.1) for patients in MSC group and 27.4% (18.3–37.3) for those in the control group (HR 2.85, 95% CI 1.40–5.80; *P* = 0.003; Fig. 2B).

The 5-year mortality associated with cGVHD was 6.9% (95% CI 1.0–12.8) for patients in the MSC group and 17.2% (7.4–27.0) for those in the control group (HR 0.42, 95% CI 0.14–1.24; *P* = 0.118; Fig. 2C).

### Survival and relapse

**Fig. 3:**
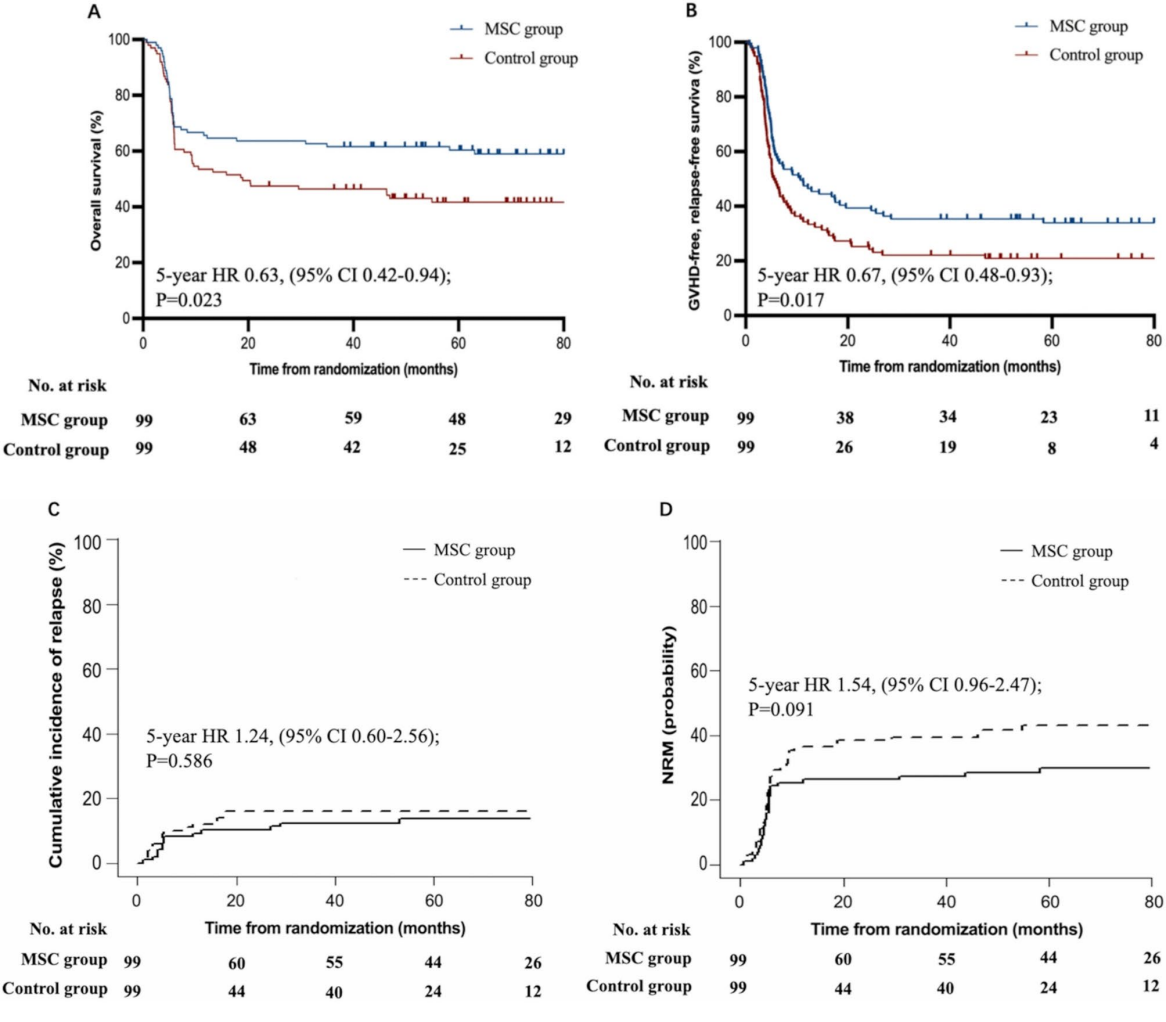
Overall survival (OS) (**A**), cGVHD-free, relapse-free survival (CRFS) (**B**), cumulative incidence of leukemia relapse (**C**), and non-relapse mortality (NRM) (**D**)

In this updated analysis, the median follow-up was 39.7 months (IQR 0.60–98.9). 101 (51.0%) patients survived and 97 (49.0%) died, of whom 40 (40.8%) were in the MSC group and 57 (58.2%) in the control group. The causes of death in MSC and control groups included primary disease relapse (*n* = 9 vs. 9), aGVHD (*n* = 9 vs. 14), cGVHD (*n* = 6 vs. 10), severe infections (*n* = 13 vs. 19), hemorrhagic disease (*n* = 3 vs. 2), thrombotic disease (*n* = 0 vs. 1) and secondary tumor (*n* = 0 vs. 2). The 5-year OS was 60.4% (95% CI 50.8–70.0) for patients in MSC group and 41.7% (31.9–51.5) for those in the control group (HR 0.63, 95% CI 0.42–0.94; *P* = 0.023; Fig. 3A). The median OS of the MSCs group was reached, but the median OS of the control group was not reached. The 5-year CRFS was 33.9% (95% CI 24.5–43.3) versus 20.9% (12.9–28.9) (HR 0.67, 95% CI 0.48–0.93; *P* = 0.017; Fig. 3B) for patients in MSC group and control group, respectively.

At the date of statistical analysis (Dec 31, 2023), 13 (13.1%) patients in the MSC group and 16 (16.2%) in the control group relapsed. The median time to relapse after study treatment was 5.3 months (IQR 2.1–53.2) for patients in the MSC group and 4.8 months (1.8–27.4) for those in the control group (*P* = 0.21, Mann-Whitney U test). The 5-year cumulative incidence of relapse was 13.6% (95% CI 7.6–21.3) for patients in the MSC group and 16.0% (9.5–23.9) for those in the control group (HR 1.24 95% CI 0.60–2.56; *P* = 0.568; Fig. 3C). The 5-year non-relapse mortality was 30.0% (95% CI 21.1–39.4) for patients in the MSC group and 42.7% (32.6–52.3) for patients in the control group (HR 1.54, 95% CI 0.96–2.47; *P* = 0.091, Fig. 3D).

### Correlative studies

To explore the possible mechanism of MSCs reducing the incidence of cGVHD in patients receiving MSCs treatment for SR aGVHD, we evaluated the differences of T, B-lymphocyte subsets and sjTRECs in the patients between two groups by flow cytometry and real-time PCR. Eighty-two patients in the MSC group and 71 patients in the control group were included in this mechanistic study, which excluded patients who received DLI as prevention or treatment for relapse within 1 year after allo-HSCT (6 vs. 7), patients with protocol crossovers (0 vs.8) and patients with data missing (11 vs.13).

**Fig. 4:**
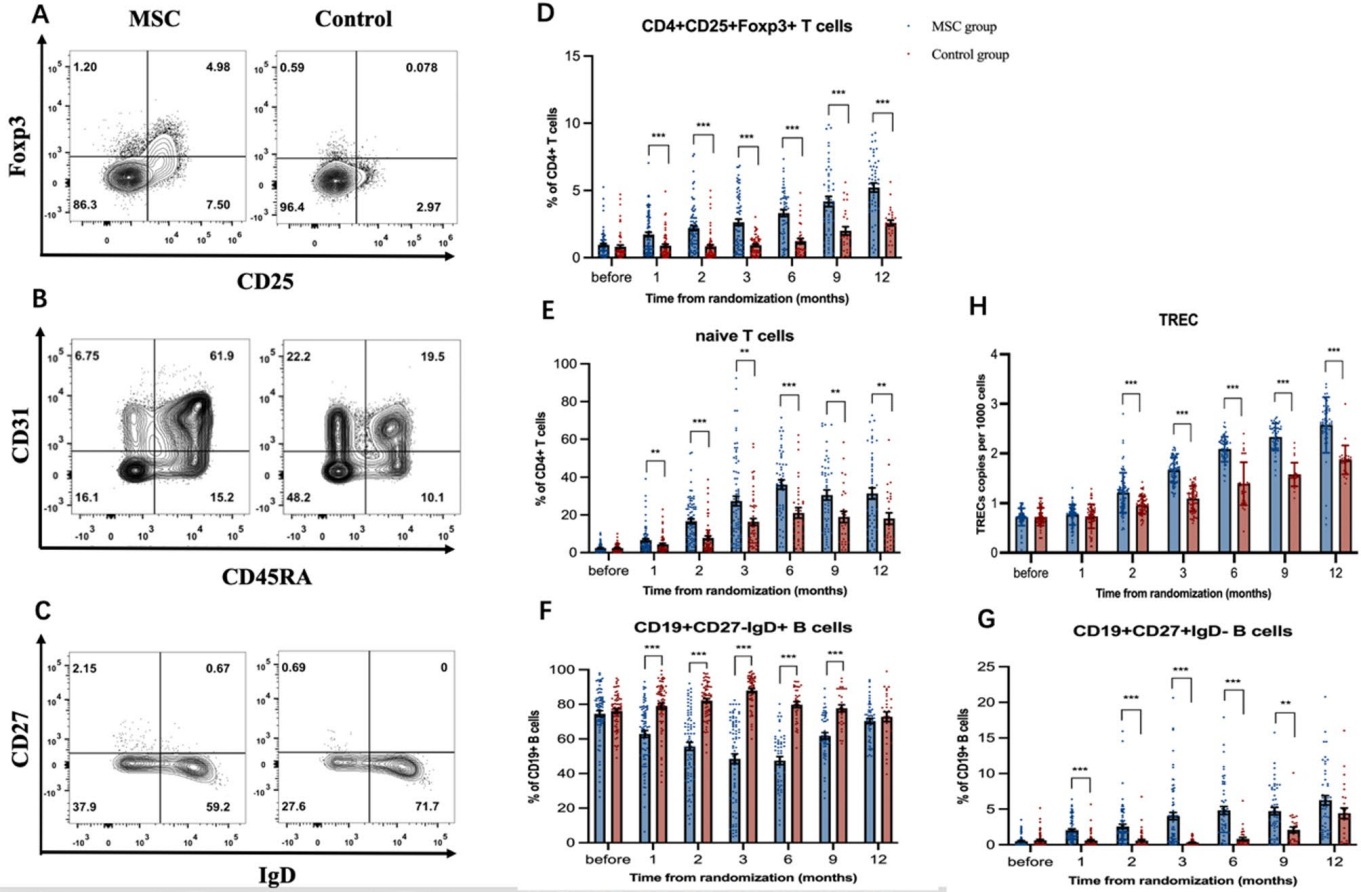
The changes of lymphocyte subsets and signal joint T cell receptor excision DNA circles (sjTRECs) in the MSC and control groups. Representative plots of the flow cytometry analysis (**A-C**). The frequencies of Tregs and naïve T cells are shown as percentage of CD3 + CD4 + T cells (**D-E**), and the frequencies of naïve B cells and memory B cells are shown as percentage of CD19 + B cells (**F-G**). The levels of sjTREC (**H**). *P < 0.05, **P < 0.01; *** *P* < 0.001. Error bars show mean +/- SEM

The frequencies of CD3^+^T cells, CD3^+^CD4^+^T, CD3^+^CD8^+^T, and CD19 + B cells among lymphocytes were not significantly different between MSC and control groups after study treatment (*P* > 0.05, Supplemental Fig. 1A-C). Further analysis of T-lymphocyte subsets indicated that the frequencies of CD4^+^CD25^+^FoxP3^+^ T regulatory cells (Tregs) in MSC group were significantly higher than those in the control group at 1, 2, 3, 6, 9 and 12 months after study treatment (*P* < 0.001; *P* < 0.001; *P* < 0.001; *P* < 0.001; *P* = 0.001; *P* < 0.001; Fig. 4D). The frequencies of CD4^+^CD45RA^+^CD31^+^ naïve T cells in the MSC group markedly increased from 1 month to 12 months after study treatment, compared with the control group (*P* = 0.022; *P* < 0.001; *P* = 0.001; *P* < 0.001; *P* = 0.008; *P* = 0.005; Fig. 4E).

Further analysis of B-lymphocyte subsets showed that the frequencies of CD19^+^CD27^−^IgD^+^ naïve B cells were significantly lower in the MSC group than those in the control group from 1 month to 9 months after study treatment (*P* < 0.001; *P* < 0.001; *P* < 0.001; *P* < 0.001; *P* < 0.001; Fig. 4F). The frequencies of CD19^+^CD27^+^IgD^−^ memory B cells were markedly higher from 1 month to 9 months in the MSC group than those in the control group (*P* < 0.001; *P* < 0.001; *P* < 0.001; *P* < 0.001; *P* = 0.001; Fig. 4G).

The median level of sjTREC in the MSC group showed a significant increase compared with that in the control group from 2 months after the study treatment, and these increases continued until 12 months after the study treatment (Fig. 4H).

## Discussion

Our earlier report of this randomized phase 3 trial after a median follow-up of 16.0 months showed that the incidence and severity of cGVHD reduced in SR aGVHD patients undergoing MSCs treatment, but significant survival benefit was not produced at the evaluation point of 3 years after randomization [17]. Meanwhile, the mechanism of MSCs reducing cGVHD has not been elucidated. The current long-term, post-hoc analysis has substantiated and further elaborated the finding that MSC could reduce the morbidity and mortality of cGVHD in aGVHD patients after a prolonged follow-up. More importantly, it translated to significant benefits of 5-year OS and CRFS. The mechanism of MSCs reducing cGVHD was through the thymus pathway.

GVHD remains the most common complication and a major cause of mortality following allo-HSCT, and cGVHD has also a detrimental effect on quality of life [1, 2, 4, 8]. Currently, aGVHD is regarded as the most powerful predictor of subsequent cGVHD [38, 39]. More than 60% of aGVHD patients proceed to cGVHD [39, 40]. In this prolonged follow-up study, our results indicated that MSCs could reduce the incidence of cGVHD evolved from SR aGVHD compared with the control group, in which our 1-year and 5-year cumulative incidence of cGVHD were respectively 35.0% vs. 49.7% and 42.0% vs. 67.1%. These extended follow-up results confirm our previous short-term study results that MSCs could reduce the incidence of cGVHD in aGVHD patients.

Currently, SR aGVHD is associated with a high mortality risk, with the 6-month survival estimated across 25 studies was 49% and the 2-year survival was 17% [41, 42]. ATG, etanercept, and basiliximab as treatments for SR aGVHD showed little to no improvement in OS [43–45]. Recently, some studies demonstrated that ruxolitinib for SR aGVHD treatment had a favorable response, and a favorable response translated to advantageous survival [45, 46]. However, the REACH3 study, a prospective randomized controlled trial showed that mortality was similar in the ruxolitinib group and the best available therapy group [33]. Our earlier report of a randomized phase 3 trial showed no significant survival benefit on MSCs treatment for SR aGVHD. However, through this long-term follow-up, 5-year OS and CRFS for patients in the MSC group were significantly higher than those in the control group, which was related to better aGVHD response and lower cGVHD morbidity and mortality.

The pathophysiological mechanism of cGVHD remains poorly understood. The risk of cGVHD increases when patients have the antecedent aGVHD, which is thought to be associated with thymic injury and dysregulated B-cell and T-cell immunity. Thymic damage caused by aGVHD impairs negative selection, permitting autoreactive donor-derived T cells to target recipient tissues, and then leads to cGVHD development [4, 12, 47]. MSCs are multi-potent stem cells that play an important role in immunomodulation and tissue repair, including thymus. In murine models, some studies including ours found that MSCs could home to GVHD-damaged thymus and restore thymic function by increasing thymocytes and thymic Tregs as well as promoting the quantity and maturity of thymic epithelial cells (TECs) [47–49]. However, a central dilemma in humans for estimating thymic function is the lack of quantitative and noninvasive markers. Until TRECs are found as the markers for estimating the number of newly generated T cells, their concentrations in peripheral blood can be used to estimate thymic output [50]. Moreover, a substantial number of studies have demonstrated that strong positive correlations of the sjTREC level and frequencies of CD31 + CD4 + naive T cells in the peripheral blood of humans have been described [51, 52]. The expression of sjTREC in CD31 + CD4 + naive T cells was similar to thymocytes, which strongly supports the CD31 + CD4 + naive T cells can be used for the analysis of recent thymic emigrants [51, 52]. In this study, we confirm in humans that the mechanism of MSCs decreases the incidence and severity of cGVHD through the thymus pathway, which significantly increases the level of sjTRECs and CD4 + CD45RA + CD31 + naïve T in peripheral blood.

Besides, the peripheral immune compartments of Tregs and B cells also play critical roles in controlling cGVHD. Some studies demonstrated that poor reconstitution of circulating Tregs led to the inability to suppress autoreactive and alloreactive immune cells and contributed to cGVHD [53, 54]. In this study, we found that MSCs could increase the frequency of Tregs in peripheral blood. Increased circulating Tregs included natural Tregs from the thymus and induced Tregs in the periphery, which indicated that MSCs decreased cGVHD through the thymus pathway and peripheral immune pathway. Perturbation of circulating B cell compartments, especially CD27 + memory B cells, may serve as a novel biomarker for monitoring cGVHD activity [28, 29, 55]. In this study, we found that the frequency of CD27 + memory B cells in the MSC group was higher than those in the control group, which also indicated that MSCs decreased cGVHD by the peripheral immune pathway.

Whether MSCs increase relapse or in vivo tumorigenesis remains our problem. The systematic meta-analysis and our long-term follow-up study evaluated the safety of systemic MSC administration and further confirmed that MSCs do not increase the risk of leukemic relapse or malignant transformation in recipients [56].

This study had a few limitations. First, the analyses in this extension study were post-hoc analyses. Second, concerning the mechanism study of MSCs reducing cGVHD, a few immune reconstitution data are missing due to the early death of SR aGVHD patients might result in bias. Third, the registration date of this research is September 2014, when ruxolitinib had not been recommended as the first-line choice for steroid-resistant aGVHD and had not been approved for marketing in China.

## Conclusions

This long-term follow-up study confirms that MSCs can reduce the morbidity and mortality of cGVHD and improve long-term survival and CRFS in patients with SR aGVHD undergoing MSC treatment. The mechanism of MSCs reducing cGVHD was through the thymus pathway.

## Electronic supplementary material

Below is the link to the electronic supplementary material.

Supplementary Material 1

## Data Availability

No datasets were generated or analysed during the current study.

## Abbreviations

GVHD: Graft-versus-host disease
allo-HSCT: Allogeneic hematopoietic cell transplantation
aGVHD: Acute GVHD
cGVHD: Chronic GVHD
MSCs: Mesenchymal stromal cells
SR: Steroid-resistant
DLI: Donor lymphocyte infusion
NR: No response
CR: Complete response
PR: Partial response
OR: Overall response
OS: Overall survival
NRM: Non-relapse mortality
CRFS: cGVHD-free, relapse-free survival
sjTREC: Signal joint T cell receptor excision DNA circles
ITT: Intention-to-treat
mITT: Modified ITT
HRs: Hazard ratios
CIs: Confidence intervals
Tregs: T regulatory cells
TECs: Thymic epithelial cells
