# Supplementary material for: Reduced morbidity and mortality of cGVHD in patients who received treatment with mesenchymal stromal cells for steroid-resistant aGVHD: long-term follow-up of a randomized phase 3 trial
Source: Exp Hematol Oncol. 2025 Jul 9;14:95. doi: 10.1186/s40164-025-00687-8 (PMC12243361; doi:10.1186/s40164-025-00687-8)

**Supplemental methods**

**Peripheral blood mononuclear cell separation**

Venous blood samples were collected in heparinized tubes for peripheral blood mononuclear cell (PBMC) separation. PBMCs were isolated by centrifugation using Ficoll-Paque PLUS (GE Healthcare Bio-Sciences AB, GE Healthcare, Uppsala, Sweden) and stored in aliquots in liquid nitrogen.

**Flow cytometry analysis**

Lymphocyte subtypes was analyzed using the following antibodies (all from BD Biosciences Pharmingen, San Diego, CA): CD3-allophycocyanin (APC), CD56-PE, CD4-fluorescein isothiocyanate (FITC), CD4-APC, CD8-phycoerythrin (PE)-cyanine (Cy)7, CD25-PE, Foxp3-APC, CD45RA-FITC, CD31-APC, CD19-Pacific Blue, CD27-PE and sIgD-FITC. For surface staining, cells were washed twice with phosphate-buffered saline (PBS) buffer containing 0.1% bovine serum albumin and 0.05% sodium azide, followed by incubation with fluorochrome-conjugated mAbs to cell surface markers at 4℃ in the dark for 30 min. Cell samples were washed twice and fixed in 1% paraformaldehyde before acquisition. To determine intracellular cytokine production, brefeldin A (10 mg/ml; Sigma-Aldrich) was added to cultures during the final 6h of the experiment. Following stimulation, cells were washed, fixed with 4% paraformaldehyde, permeabilized and stained for the intracellular cytokines in PBS buffer containing 0.1% saponin for 30 min at 4℃. After intracellular staining, cells were washed and resuspended in PBS. Flow cytometry was performed using a BD FACSCalibur cytometer. Lymphocytes were gated on forward and side scatter profiles and analyzed using FlowJo software (Treestar, San Carlos, CA, USA).

**Quantification of T-cell receptor rearrangement excision circles**

DNA was extracted using QIAamp DNA Mini Kit (Qiagen, Chatsworth, California, USA). sjTREC concentrations were determined by quantitative real-time PCR based on the coding sjTREC sequence using LightCycler 480 II (Roche) and primers to amplify a DNA fragment 108bp across the remaining recombination sequence 𝛅rec/𝛙𝛂 (5’-CACATCCCTTTCAACCATGCT-3’) and 5’-GGTGCAGGTGCCTATGC-3’). For quantification, a synthetic internal standard (Ingenetix, Vienna, Austria) was used. PCR reaction ran with 0.25𝜇g DNA, primers and hybridization-probe labelled with FAM-TAMRA (5’-ACACCTCTGGTTTTTGTA AAGGTGCCCAC-3’).

**Supplemental Figure**

**Supplemental Figure 1 Overall Survival and cGVHD-free, relapse-free survival (CRFS) in as-treated population**

The as-treated population consisted of 107 patients in the MSC group (99 patients plus 8 patients with protocol crossovers), and 91 patients in the control group (99 patients minus 8 patients with protocol crossovers). In the as-treated analysis, the 5-year OS was 61.4% (95% CI 52.2–70.6) for patients in MSC group and 39.8% (29.6–50.0) for those in the control group (HR 0.61, 95% CI 0.41–0.92; P=0.016, Supplemental Figure1A). In the as-treated analysis, the 5-year CRFS was 33.2% (95% CI 24.2–42.2) versus 20.6% (12.2–29.0) (HR 0.66, 95% CI 0.47–0.92; P=0.012, Supplemental Figure1B) for patients in MSC group and control group, respectively.


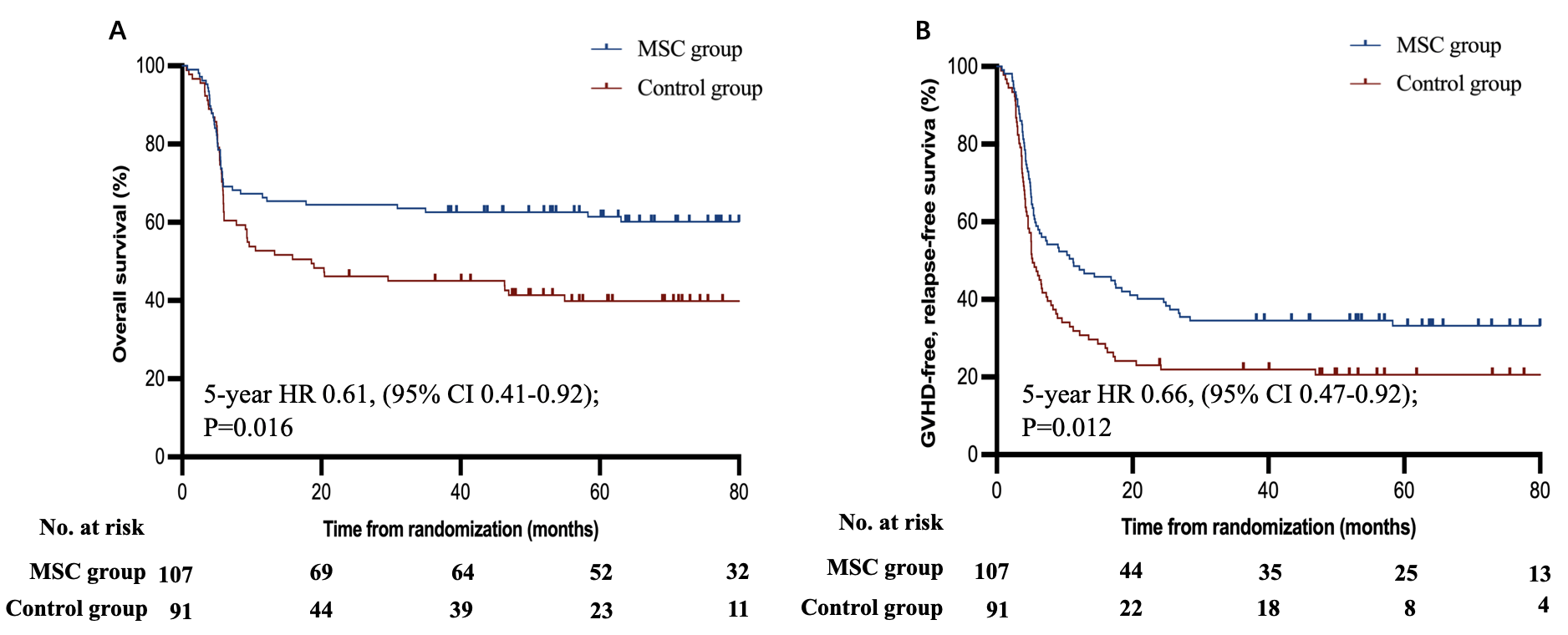


**Supplemental Figure 2 The frequencies of T- and B-cell subsets**

(A-C) Representative plots of the flow cytometry analysis; (D) The frequencies of CD3+ T cells are shown as percentage of the lymphocytes between MSC and the control groups; (E) The frequencies of CD3+CD4+ T cells are shown as percentage of the lymphocytes between the two groups; (F) The frequencies of CD3+CD8+ T cells are shown as percentage of the lymphocytes between the two groups; (G) The frequencies of CD19+ B cells are shown as percentage of the lymphocytes between the two groups. Error bars show mean +/- SEM.


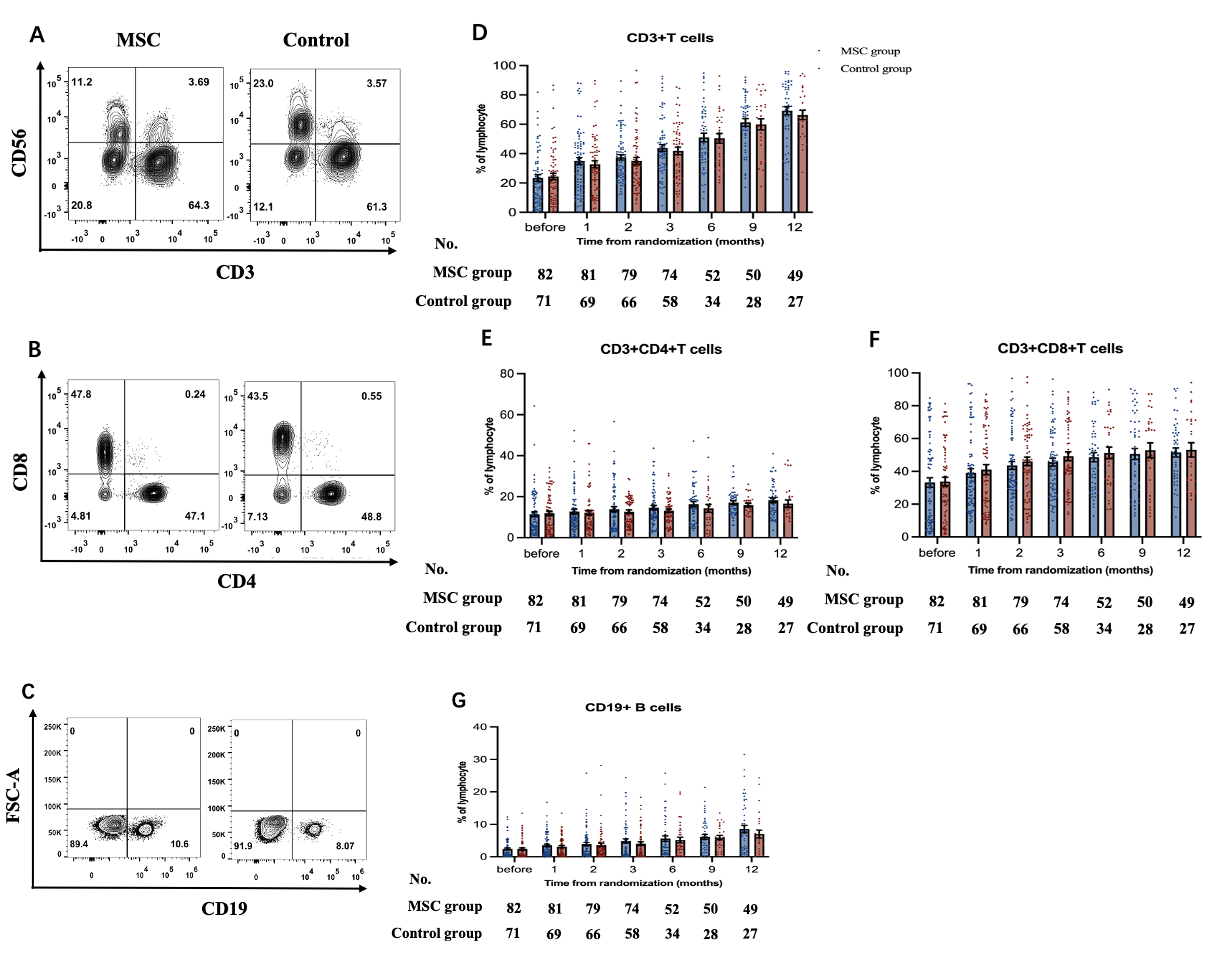

Supplement: Supplementary file 1 — Supplementary Material 1 [file 40164_2025_687_MOESM1_ESM.docx]
